# Supplementary material for: Co-design of “Baatcheet,” a peer-supported, web-based storytelling intervention for young people with common mental health problems in India
Source: Glob Ment Health (Camb). 2024 Dec 16;11:e128. doi: 10.1017/gmh.2024.148 (PMC11704371; doi:10.1017/gmh.2024.148)
Supplement: Gonsalves et al. supplementary material [file S2054425124001481sup001.docx]

**ANNEXURE 1: YPAG MEETING OVERVIEW**

| **Meeting** | **Objectives** | **Activities** | **Timeline** |
| --- | --- | --- | --- |
| 1 | Introduce Baatcheet and the role of YPAG  Introduce mental health storytelling  Develop a logic model | Group ice-breakers  Community ground rule setting  PPT  Interactive storytelling workshop  Tearless logic model (Lien et al, 2011) | January 2023 |
| 2 | Identify changes for the existing It’s Ok To Talk website  Identify guiding principles and values of Baatcheet  Brainstorm features & functions of the Baatcheet website | MoSCoW prioritisation (Clegg and Barker, 1994) of existing IOTT features  Brainstorm and ranking guiding principles and values  Group discussion to brainstorm features and functions of Baatcheet | April 2023 |
| 3 | Familiarise with the concept of peer support  Identify preferences for peer counsellor qualities, type of support and delivery format  Gather feedback on call for stories | Case study based scenarios  Questionnaire  Ranking formats of delivery  Brainstorm on peer-counsellor challenges and benefits  Think aloud to share feedback on call for stories | May 2023 |
| 4 | Gather feedback on the website prototype  Review story based reflection questions  Finalise list of reaction buttons for the website | Demo of the website prototype  Think aloud along with discussion  Review and suggest changes to story-based reflections  Rank top 4 from a long list of reaction buttons | July 2023 |
| 5 | Share findings about peer support from co-design workshops  Identify considerations for peer counsellor recruitment, training and support during delivery  Identify recommendations for designing a call for recruitment poster of peer counsellors | Group discussion on peer counsellor role, selection criteria, training needs, incentives and provisions of support during delivery  Brainstorm a list of design considerations for the call for recruitment poster | Sept. 2023 |
| 6 | Recap Baatcheet’s progress and contributions of YPAG  Envision a scale up plan of the Baatcheet programme  Reflect on changes experienced as a youth advisor  Gather feedback on experiences of participation as a youth advisor | Presentation on Baatcheet and YPAG contributions  Sketching a big picture of vision for Baatcheet 2.0  Reflective body mapping to reflect on the changes undergone as a Baatcheet advisor and share the Most Significant Change [MSC]  Feedback form to share about experiences of participation and areas of improvement | March 2024 |

**ANNEXURE 2: CONSULTATION WORKSHOP AGENDA**

**DATE:** 4TH JULY 2023

**TIMINGS:** 9.30 AM-4.30 PM

**VENUE:** MAPLE ROOM, INDIA HABITAT CENTRE, NEW DELHI, INDIA

| **Time** | **Agenda** | **Moderator / Presenter** |
| --- | --- | --- |
| 9 - 930 AM | Tea & welcome | |
| 930 - 10 AM | Attendee introductions | Pattie Gonsalves |
| 10 - 1030 AM | Workshop objectives & background | Pattie Gonsalves |
| 1030 - 1040 AM | Opening remarks | Daniel Michelson |
| 1040 - 11 AM | Role of Youth Advisors  Initial insights from our research | Priyambada Kashyap  Dhriti Mittal  Aarushi Khan |
| 11 - 1130AM | Baatcheet prototype | Manek D’Silva |
| 1130 AM - 12 PM | Break | |
| 12 - 1 PM | ‘Paints, pillows and pain”  Story sharing by Sonaksha followed by reflection and Q&A | Sonaksha |
| 1 - 2 PM | Lunch | |
| 2 -4 PM | Design thinking: ‘How might we?’ | Facilitated group work |
| 4 - 430 PM | Closing | Pattie Gonsalves |

**ANNEXURE 3:**

**CONSULTATION WORKSHOP SUMMARY**

RESPONSES FROM GROUP DESIGN ACTIVITY “HOW MIGHT WE”

**Stage 1: Development of a logic model**

**Attendees**

The consultation workshop included 20 mixed stakeholders and five members of the development team.

**Activity**

A day-long consultation workshop was conducted to gather feedback on an initial intervention outline prepared by the study team and YPAG. Attendees were divided into smaller groups of 5-6 individuals. Each group engaged in a structured feedback activity using the ‘MoSCoW’ (Must have, Should have, Could have and Won’t have) prioritization tool (Clegg & Barker, 1994).

**Responses from Group Design Activity**

**Group 1:** How might we encourage young people to use the Baatcheet website/ participate in the programme?

| **Must have** | **Should have** | **Could have** | **Won’t have** |
| --- | --- | --- | --- |
| Include testimonials to showcase programme benefits.  Begin with telling stories offline, then move online.  Consider contributor privacy carefully from the beginning of the programme.  Emphasise that anyone can contribute regardless of writing skills.  At the start of the story, provide a reflective question  . | Implement psychoeducational workshops in schools, involving students and parents.  Provide a mood check-in option as part of the website that leads the user toward a page with available resources to support participants' mental health concerns.  Offer optional anonymity for contributors. | Make the website feel personal and relatable, as if one is interacting with a real person. |  |

**Group 2:** How might we enable schools and colleges to become spaces where mental health storytelling by students is encouraged and safe?

| **Must have** | **Should have** | **Could have** | **Won’t have** |
| --- | --- | --- | --- |
| Conduct de-stigmatisation workshops in different schools, collaborating with existing systems (e.g., with the Delhi government).  Host and organise events such as youth mental health festivals at schools and colleges, incorporating visual arts, theatre, drama, and short films, all centred around mental health storytelling.  Establish liaisons with school counsellors, incorporating Baatcheet to address mental health challenges within educational settings. | Offer tailored/need based workshops addressing specific concerns like bullying and exam stress.  Incorporate the feedback of stakeholders to develop an intervention that addresses the long-term needs of the community.  Use multimedia tools, school spaces, and communication materials to share about Baatcheet and its impact.  Share success stories of others who have benefited from similar approaches, fostering a sense of community support. | Introduce an anonymous sharing method (e.g., drop boxes) for students to share struggles and stories.  Integrate mental health concepts into extracurricular activities/ classes like craft, drama, and dance to promote mental health awareness.  Establish student-led mental health clubs to organise Baatcheet-related projects, events, and awareness campaigns. |  |

**Group 3:** How might we encourage young people to feel motivated to share their story via platforms like Baatcheet?

| **Must have** | **Should have** | **Could have** | **Won’t have** |
| --- | --- | --- | --- |
| Ensure ownership of stories is shared with contributors.  Separate the writing process from the publishing process.  Highlight the potential benefits of self-expression and sharing stories. | Display real people's short stories upfront on the website.  Ensure clear requests for any user data that is collected.  Develop outreach programs tailored to institutional spaces and underserved communities.  Engage local, regional, and community stakeholders to encourage participation.  Incorporate narrative therapy principles for effective story construction.  Clearly differentiate between user reflections and published stories. | Enable multimedia format submissions for added engagement.  Share testimonials from those who shared stories to understand the impact. | Do not mandate story sharing and avoid promoting it as a necessary feature.  Eliminate direct publication options to maintain controlled content quality.  Avoid glorifying sharing; highlight both risks and benefits.  Do not use resource-intensive and long editorial procedures. |

**Group 4:** How might we motivate young people (including those with lived/living experience) to support other young people as ‘peer supporters’ through Baatcheet?

| **Must have** | **Should have** | **Could have** | **Won’t have** |
| --- | --- | --- | --- |
| Provide the option for the user to select preferred peer supporters based on specific criteria.  Emphasise the importance of community support.  Create, establish and promote a community of young individuals who need mental health support.  . | Organise events like "The Human Library" sessions where participants can hear stories from those they would not normally meet in an effort to break down prejudices and stigma.  Invite potential peer supporters from schools and community-based organisations.  Use more accessible terms than "peer supporter".  Incorporate use of arts-based approaches such as art and movement-based therapy exercises for engaging young people. | Consider offering honorariums or certificates of experience.  Add peer supporters' stories on the website. | Avoid personal biases, judgments in language, and any unnecessary self-disclosure by peer supporters.  Avoid unrealistic commitments or assurances by peer supporters. |

**Group 5:** How might we reduce the access barriers that young people may face in participating in the Baatcheet programme?

| **Must have** | **Should have** | **Could have** | **Won’t have** |
| --- | --- | --- | --- |
| Employ visually captivating content that resonates with users' cultural backgrounds  Ensure a swift browsing experience with a lightweight design, and offer offline access for users with limited connectivity.  Partner with educational and healthcare institutions to effectively distribute awareness materials.  Prioritise user privacy through informed consent and robust data protection measures.  Boost engagement by organising school competitions and offering incentives. | Promote the website by emphasising the participation of users from different parts of India, especially those outside of Delhi, to show that the program positively impacts a wide range of people.  Showcase real-life success stories from platform users.  Improve accessibility through night mode, read-aloud option, and a concise text dictionary for easier comprehension.  Enhance inclusivity with speech-to-text functionality and alternative image text descriptions. | Implement a comprehensive strategy to raise broad awareness and support for mental health.  Provide a curated list of effective self-coping strategies.  Enable users to track their moods post-engagement | Provide content and features without paywalls.  Deliver uninterrupted user interactions by abstaining from using advertisements. |

**ANNEXURE 4: CO-DESIGN FOCUS GROUP DISCUSSION (FGD) GUIDE**

**Welcome**:

- Introduce moderator and co-moderator

*Good afternoon and welcome to our session today. Thanks very much for taking the time to talk with us about a school mental health initiative. My name is <Name> and <Name> will be assisting me today. We are both based at Sangath, a mental health research organisation in New Delhi.*

- Our topic is..

*Our organisation was recently awarded a research grant to build a web-based mental health support programme called “Baatcheet” that uses stories of young people to reduce anxiety and depression among youth aged 16-24 years. You will be participating in 4 workshops over the next 6 months. We really want to know what you like, what you don’t like and how a programme like this could fit into your school/college/CBO. We also want to learn about possible challenges that could come up in delivering a programme like this so that we can work around these. We are having discussions like this with a few other groups too and will compile these learnings together.*

- The results of this discussion will be used for…

*The larger aim through this programme is to build a storytelling programme which can help young people with mental health problems especially depression and anxiety. What you share with us today will be very useful in helping us decide what types of information and stories Baatcheet will contain, what it might look like and how it can be used most easily by young people.*

- You have been invited/selected because…

*You have been invited because you expressed an interest in participating or because your school/college/CBO has nominated you.*

**Guidelines for participation:**

- There are no right or wrong answers, only different points of view
- We will be recording this discussion, on student/person speaking at a time
- We are on a first name basis
- You don’t need to agree with others/everyone, but must listen to everyone respectfully
- Be curious and open to sharing

*There are no wrong answers but rather differing points of view. Please feel free to share your point of view even if it differs from what others have said. Keep in mind that we're just as interested in negative comments as positive comments, and at times the negative comments are the most helpful.*

*You've probably noticed the microphone. We're tape recording the session because we don't want to miss any of your comments. People often say very helpful things in these discussions and we can't write fast enough to get them all down. We will be on a first name basis, and we won't use any names in our reports. You may be assured of complete confidentiality. The information you share with us will help our team design our programme.*

*Well, let's begin. We've placed name cards on the table in front of you to help us remember each other's names. Let's find out some more about each other by going around the table.*

**Question guide**

**Ice breaker/warm up question**

1. Tell us your name, preferred pronouns if you’d like, where you are from and what do you do to take care of your mental health?

**Storytelling**

1. Have you ever heard someone else’s personal experience or story about having a MH concern? (Perhaps read, watched or listened to) If yes,
   - Whose experience(s) did you hear?
   - Where did you hear it/these stories?
   - Did the story impact you? If so, how? (What difference (if any) has it made to (you or someone you know) to hear their experience (especially for problems such as depression and anxiety)?)
   - What do you think of learning about mental health problems through the personal stories of people with mental health problems (such as depression and anxiety)?

**Mental health story content**

1. Which messages were most helpful to you in your own understanding of MH or others'?
   - Is there anything you have remembered / held on to / that stood out to you from someone’s story?
2. Was there anything in the story that you did not like?
   - Is there anything you found difficult to understand from what they might have shared?

**Storytelling platforms**

1. If you or someone you know was going through a mental health difficulty, where would you/have you look(ed) for mental health information?
2. Currently, if you were to search for stories about mental health experiences, where would you go? (E.g., online)
   - Which types of online platforms would you feel safe on? (E.g., Instagram, mental health or youth organisational websites, storytelling websites, YouTube influencers, celebrities)
   - Whose stories would you want to see?
   - Is there a particular media format (e.g., video, text, audio, etc) you would prefer over others?

Activity What themes and messages would be especially helpful to hear about from stories/experiences? (Choose as many as you like)

- normalising that many young people have mental health problems
- that it’s OK to struggle
- information on why depression/anxiety happens
- the symptoms of depression/anxiety
- messages of hope
- how recovery happens
- experiences of opening up to friends or family
- how to ask for help
- how to find professional help
- what happens in counselling/therapy
- what it is like to take medication
- specific coping techniques such as relaxation or mindfulness or problem solving
- how to handle negative thoughts and feelings
- how to stay physically active when experiencing depression or anxiety
- how to find meaningful activities to make you feel better
- how to stay connected to others
- Other (please mention)

**Support/guidance**

1. Would some kind of 1-1 direct support from another person be helpful in participating in a mental health storytelling programme? E.g., in finding the right story; checking in to see how you’ve been doing; if you have been able to make use of things you have learned
2. Who would the person be (E.g., peer/friend, teacher, counsellor, etc)? And why?
   - Is it important that they are similar in age or from the same college/school?
   - Is it important that they have had a lived experience/prior experience of mental health problems such as depression or anxiety?
   - What kind of support do you expect from them?
3. If support came from a peer, what are the advantages and disadvantages?

**Telling one’s own story**

1. Do you think the programme should ask participants to share their own stories too? How do you think participants would feel about this?
   - If yes, when would a good time to share be? At the start of a programme like Baatcheet or as part of it or at the end?
   - Should this be totally optional/flexible?
   - Do you think allowing participants to share anonymously will help them to share their story?
2. Do you feel it is helpful to disclose/share one’s personal experience/story of going through a mental health problem
   - for yourself/your own mental health?
   - to help others?

**ANNEXURE 5: CO-DESIGN WORKSHOPS OVERVIEW**

**Workshop 1: Identifying youth preferences for Baatcheet website**

Objective(s):

1. To understand youth preferences regarding the features, story contents and functionality of the Baatcheet website.
2. To identify features to enhance engagement with Baatcheet.

Duration: 1.5-2 hours

Activities:

|  | **Agenda** | **Activity and description** | **Output(s)** |
| --- | --- | --- | --- |
| 1 | Introduction to the It’s Ok To Talk website | Demonstration of the website followed by individual review. | Build group familiarity of a storytelling website. |
| 2 | Gather youth preferences for features, story content, and functionality of the Baatcheet website. | Design studio: Breakout groups create paper prototypes of the website, focusing on presentation, categorization, and filtering of stories, and identifying strategies to enhance interactivity | Web-app aspects/features  Design elements  Intervention engagement preferences |

**Workshop 2: Identifying youth preferences for optimising peer delivery**

Objective(s):

1. To understand the cultural acceptability of the intervention delivery by peer supporters
2. To identify preferences for peer support (information, peer supporter qualities, peer support type/format and delivery)

Duration: 1.5 hours

Activities:

|  | **Agenda** | **Activity and description** | **Output(s)** |
| --- | --- | --- | --- |
| 1 | Introduction to peer support | Short information session about peer support in mental health interventions | Build group familiarity of peer delivery of mental health interventions |
| 2 | Identify preferences for peer counsellor qualities, characteristics and role specification | Case-vignettes with questions about peer supporter qualities and role  Questionnaire assessing comfort level in seeking support from peers of varying ages, educational backgrounds, and experiences | List of preferences for peer supporter qualities, characteristics and support type |
| 3. | Identify preferences for peer delivery format | Ranking a list of options for delivery formats along with reasons for most and least preferred option | Preferences for delivery format |
| 4. | Identifying anticipated challenges and benefits of peer support | Group discussion on benefits, challenges and potential steps to overcome these challenges. | List of anticipated challenges and benefits of peer support, and strategies to address challenges |

**ANNEXURE 6: GUIDELINES FOR STORY TAGGING**

**CONTEXT**

These guidelines have been prepared for tagging story and narrator characteristics for the Baatcheet intervention. All included stories  will  contain these tags which will be coded into the backend of the website and displayed via filters for selection and hashtags to the Baatcheet users. These guidelines have been modelled on the INCRESE (Inventory of Characteristics of Recovery Stories) guidelines prepared by Llewellyn-Beardsley et al (2019).

**SECTION 1: STORY ELIGIBILITY**

This section codes whether a story can be included in the Baatcheet programme. A story can be included if all four sections below are ticked.

| **#** | **Item** |
| --- | --- |
| 1 | Narrator’s own lived experience of mental health problems, especially depression and anxiety. |
| 2 | The story includes a description of adversity/struggle. |
| 3 | The story includes a description of hope, strength, or successes in relation to the mental health problem. |
| 4 | The story was submitted as text, video, audio or artwork. |

**SECTION 2: STORY CHARACTERISTICS**

Select the relevant option.

| **#** | **Item** | **Description** | **Tags** |
| --- | --- | --- | --- |
| 1 | Media format | The story is in one or more of the following media formats: blog, poetry, audio, video, art. | Article  Poem  Video  Art |
| 2 | Estimated reading time | The estimated time to read the story. | 1-99 minutes (added as an integer) |

**SECTION 3: NARRATOR CHARACTERISTICS**

Choose a specific response only if the narrator explicitly identifies that response

| **#** | **Item** | **Description** | **Tags** |
| --- | --- | --- | --- |
| 1 | Age | The age of the narrator at the time of submission. | Below 16  16-18  19-21  22-25  26-30  Above 30 |
| 2 | Gender | The gender that the narrator identifies with. Gender may include: Male, female, transgender, non-binary, not mentioned. | Male  Female  Trans  Non-binary  Prefer not to say |
| 3 | Location | The city the narrator lives in. E.g., Delhi, Mumbai, Chennai, etc. | <City Name, State Name> |
| 4 | Communities | If the narrator has described  their marginalised experiences such as sexuality, caste, religion, etc. | LGBTQIA+  Person with disability  Dalit, Bahujan or Adivasi  Religious minorities  Not applicable |

**SECTION 4: NARRATIVE CONTENT**

Select one item per tag type that best characterises the significant parts of the story

| **#** | **Type** | **Item** | **Description** |
| --- | --- | --- | --- |
| 1 | Mental health problem | Depression |  |
| 2 |  | Anxiety |  |
| 3 | Problem | Body image | Perception of one's physical appearance and self-image |
| 4 |  | Break up | End of a romantic relationship |
| 5 |  | Education | Challenges related to academia or education |
| 6 |  | Suicide | Thoughts, feelings or actions to end one’s life |
| 7 |  | Stigma | Negative attitudes, beliefs, and stereotypes leading to discrimination and exclusion of a particular group or condition |
| 8 |  | Abuse | Characterises physical or psychological maltreatment towards others |
| 9 |  | Trauma | Emotional response to distressing event |
| 10 |  | Loneliness | A feeling of being socially or emotionally disconnected, typically accompanied by a sense of isolation or solitude |
| 11 |  | Overthinking | Over dwelling on thoughts |
| 12 |  | Bullying | Repeated aggression to harm or intimidate on others involving power imbalance |
| 13 |  | Self harm | Intentional act of inflicting physical harm to oneself to cope with emotions or regain control |
| 14 |  | Loss (Grief) | Deep sorrow or mourning, often associated with the loss of someone or something significant |
| 15 |  | Addiction | Addiction involves craving for something intensely, loss of control over its use, and continuing involvement with it despite adverse consequences |
| 16 |  | Relationship | Positive or negative experiences related to romantic relationships |
| 17 |  | Burnout | Burnout is a state of emotional, physical, and mental exhaustion caused by excessive and prolonged stress |
| 18 |  | Violence | behaviour which harms or damages somebody/something physically |
| 19 |  | Societal pressure | External influences or expectations from society that can exert stress or influence behaviour, choices, or norms |
| 20 |  | Symptoms | An indication or sign of illness |
| 21 |  | Discrimination | Treating one person or group worse than others |
| 22 |  | Family | Positive or negative experiences related to family |
| 23 |  | Loss | The experience of losing someone or something significant |
| 24 |  | Self image | the idea one has of one's abilities, appearance, and personality |
| 25 |  | Hospitalisation | Positive or negative experiences of voluntary or involuntary hospitalisation |
| 26 |  | Blame | To think or say that a certain person is responsible for something bad that happened |
| 27 |  | Post-traumatic stress disorder (PTSD) | A mental health problem which can develop after experiencing traumatic events |
| 28 |  | Relapse | The return of an illness after improvement |
| 29 |  | Migration | The movement of a person from one place to another |
| 30 | Turning point | Hope | A positive outlook or expectation for the future, often accompanied by a belief in favourable outcomes |
| 31 |  | Acceptance | Change after e.g. an increase in confidence, growth in self-awareness, emotional release, moving away from internalised stigma. This may occur through own learning/inner work, involvement with support or other groups, or counselling/ therapy. |
| 32 |  | Seeking help | Reaching out to others for support, e.g., family, friends, professionals |
| 33 |  | Spirituality | Personal search for meaning, purpose, or connection to something beyond oneself |
| 34 |  | Advocacy | Action to create a change in the existing systems |
| 35 | Coping strategies | Social support | Presence or absence of a network of individuals providing assistance, encouragement, or emotional support |
| 36 |  | Self care | Strategies or techniques to manage or deal with challenging or stressful situations  (self help strategies, self love, affirmations, journaling) |
| 37 |  | Therapy | Seeking professional assistance, treatment and guidance in resolving mental health problems |
| 38 |  | Medication | Experience of taking medicines as a part of treatment |
| 39 |  | Rehabilitation | Restoring someone to health or normal life through training and therapy after imprisonment, addiction, or illness |

**SECTION 5: CONTENT WARNINGS**

Any content that is sensitive or can be triggering in nature

| **#** | **Item** | **Description** |
| --- | --- | --- |
| 1 | Bullying | Unwanted behaviour, physical or verbal (or even suggested), that makes someone feel uncomfortable, humiliated, or mentally distressed |
| 2 | Self-harm | Direct experiences or witnessing of e.g. deliberate injury or harm to oneself, neglect of self, alcohol or substance misuse |
| 3 | Suicide | Thoughts, feelings or actions to end one’s life |
| 4 | Abuse | Direct experiences or witnessing of any form of sexual, physical or emotional abuse, neglect, domestic violence or acts of sexual violence |
| 5 | Loss (Grief) | Mention of someone’s death in the narrative |
| 6 | Discrimination | Direct experiences, witnessing of or reference to discriminatory actions, behaviours, or decisions on the basis of e.g., caste, sexuality, religion, etc |

**SECTION 6: QUESTION BUCKET CATEGORIES/COLLECTIONS**

Choose the closest matching response for each item. Many narratives may fit more than one of these responses. Please give your best estimate as to the response which best fits the narrative overall.

| **#** | **Item** |
| --- | --- |
| 1 | What does depression or anxiety feel like? |
| 2 | How does recovery happen? |
| 3 | What does professional mental health help look like? |
| 4 | What are tips to manage mental health problems? |
